# The added values of ^18^F-FDG PET/CT in differentiating cancer recurrence and osteoradionecrosis of mandible in patients with treated oral squamous cell carcinoma

**DOI:** 10.1186/s13550-023-00965-8

**Published:** 2023-04-03

**Authors:** Nai-Ming Cheng, Chien-Yu Lin, Chun-Ta Liao, Din-Li Tsan, Shu-Hang Ng, Tzu-Chen Yen

**Affiliations:** 1 Department of Nuclear Medicine and Molecular Imaging Center, Chang Gung Memorial Hospital, Linkou, Chang Gung University College of Medicine, No. 5, Fu-Shin St., Kueishan District, Taoyuan City, 333 Taiwan; 2 Department of Nuclear Medicine, Chang Gung Memorial Hospital, Keelung, Keelung, Taiwan; 3 Department of Radiation Oncology, Chang Gung Memorial Hospital, Linkou, Chang Gung University College of Medicine, Taoyuan, Taiwan; 4 Department of Otolaryngology – Head and Neck Surgery, Chang Gung Memorial Hospital, Linkou, Chang Gung University College of Medicine, Taoyuan, Taiwan; 5 Department of Radiation Oncology, Chang Gung Memorial Hospital, Keelung, Taiwan; 6 Department of Diagnostic Radiology, Chang Gung Memorial Hospital, Linkou, Chang Gung University College of Medicine, Taoyuan, Taiwan

**Keywords:** PET/CT, Total lesion glycolysis, Oral cancer, Osteoradionecrosis, Recurrence

## Abstract

**Background:**

Osteoradionecrosis (ORN) of the jaw requires a differential diagnosis to exclude cancer recurrence. Here, we sought to develop a scoring system comprising ^18^F-FDG PET/CT parameters for distinguishing between the two conditions in patients with oral squamous cell carcinoma (OSCC).

**Methods:**

The study consisted of 103 OSCC patients with suspected ORN of the jaw. All participants underwent ^18^F-FDG PET/CT imaging within 6 months of diagnostic histopathology. Following extraction of PET parameters, we identified clinical and imaging predictors of mandibular recurrence-free survival (MRFS) using receiver operating characteristic curve analysis and multivariate Cox regression models.

**Results:**

The results of histopathology revealed mandibular cancer recurrence in 24 patients (23.3%). Multivariate Cox regression analyses identified an age at diagnosis ≤ 52 years (*P* = 0.013), a location of the SUVmax voxel with soft tissue predominance (*P* = 0.019), and mandibular total lesion glycolysis (TLG) > 62.68 g (*P* < 0.001) as independent risk factors for MRFS. A scoring system was devised with scores from 0 (no risk factor) to 3 (presence of all three risk factors). High-risk patients with a score of 2–3 compared with score of 0–1 had a significantly higher likelihood of mandibular cancer recurrence (hazard ratio: 32.50, 95% confidence interval: 8.51–124.18, *P* < 0.001). The scoring system had a sensitivity of 87.50%, a specificity of 82.28%, and an accuracy of 83.50% for identifying mandibular cancer recurrence.

**Conclusions:**

The scoring system of our study is clinically useful for identifying mandibular cancer recurrence in patients with suspected ORN of the jaw.

**Supplementary Information:**

The online version contains supplementary material available at 10.1186/s13550-023-00965-8.

## Introduction

While being an integral part of the multidisciplinary management of oral squamous cell carcinoma (OSCC), radiotherapy (RT) might cause various complications—of which mandibular osteoradionecrosis (ORN) is one of the most feared. Published data suggest that the prevalence of this condition following RT varies from 2 to 9%, with the main risk factors being age > 55 years [1, 2], active smoker at diagnosis [3] and RT doses > 60 Gy [4]. In general, ORN can be defined as an area of exposed devitalized irradiated bone that fails to heal over a period of three months without signs of recurrent or residual malignancy [5, 6]. The pathogenesis of ORN is complex and includes local inflammation, damage to vascular supply as a result of surgery or obliterative endarteritis, and altered bone healing accompanied by an increased susceptibility to infections [7–9].

Due to distinct clinical management, mandibular ORN requires a differential diagnosis to exclude tumor recurrence. Although ^18^F-FDG PET/CT imaging is widely used in the evaluation of therapeutic outcomes and post-treatment surveillance of patients with OSCC [10], there are limited data on its potential usefulness for distinguishing between mandibular ORN and cancer recurrence. On analyzing a sample of 37 patients with head and neck malignancies arising from different anatomical sites, Meerwein and coworkers [11] have previously shown that a combination of three parameters—i.e., a low maximum standardized uptake value (SUVmax), the location of SUVmax voxel within the bone, and the presence of a pathological fracture—was independently associated with ORN. However, there was a significant overlap of SUVmax measurements between recurrent cancer and ORN [12, 13]—a finding attributable to the increased FDG uptake elicited by both hypoxia and bone tissue inflammation [14, 15]. In recent years, there has been significant interest in extracting quantitative information from PET images, i.e., radiomics, to improve the prediction accuracy of clinical outcomes [16–18]. In this scenario, we designed the current retrospective study to examine whether ^18^F-FDG PET/CT functional parameters may be clinical useful for distinguishing between mandibular ORN and cancer recurrence in patients with OSCC.

## Patients and methods

### Study participants

The present retrospective study was conducted using reviewing chart records from patients with OSCC who had been diagnosed between April 2004 and April 2021 in the Chang Gung Memorial Hospital (Linkou, Taiwan). All participants had undergone primary treatment with curative intent—including RT with a total delivered dose > 50 Gy—and had suspected mandibular ORN clinically by chart records. Patients underwent ^18^F-FDG PET/CT imaging and histopathological work-up for the diagnosis of mandibular lesions within the subsequent six months. Patients who achieved complete remission from a previous malignancy for at least 1 year were eligible for inclusion. Subjects with persisted second primary malignancies or aged less than 20 years were excluded, as were those with evidence of metastatic disease at presentation. The study protocol was approved by the Institutional Review Board of Chang Gung Medical Foundation (approval numbers: 202102071B0). The requirement for written patient informed consent was waived due to the study design. Our study was performed in accordance with the ethical standards as laid down in the 1964 Declaration of Helsinki and its later amendments or comparable ethical standards.

### Post-treatment surveillance, staging, and data collection

According to our institutional guidelines, patients with OSCC who received primary treatment were scheduled to undergo imaging follow-up—including ^18^F-FDG PET/CT, CT, or MRI scans—every three months for the first year and every six months thereafter. Enrollment encompassed a 17-year period (2004–2021) during which different editions of the American Joint Committee on Cancer (AJCC) Staging Manual were applied; for consistency, all patients included in the study were staged according to the eighth edition of the AJCC Staging Manual [19]. Patient characteristics—including risky oral habits (i.e., lifetime smoking and alcohol use) and the date of suspected mandibular involvement—were retrospectively extracted from clinical records.

### Outcome definition

Mandibular relapse-free survival (MRFS)—defined as the time elapsed from the termination of primary RT to the date of mandibular cancer recurrence confirmed by histopathology—served as the main outcome measure. Censoring was performed on the date of the last follow-up (i.e., administrative censoring) for those without mandibular cancer recurrence.

### ^***18***^***F-FDG PET/CT acquisition***

The median time interval between PET imaging and the results of histopathology was 41 days (interquartile range: 14–93 days). Patients underwent PET/CT imaging procedures on either a Discovery ST 16 scanner (GE Healthcare, Milwaukee, WI, USA) or a Biograph mCT scanner (Siemens Medical Solutions, Malvern, PA, USA) after a 6-h fast. The injected ^18^F-FDG dose ranged between 370 and 555 MBq according to the patient’s body weight. No intravenous contrast agent was used for CT scans. Images were reconstructed using an ordered-subset expectation maximization (OSEM) algorithm (4 iterations and 10 subsets for the Discovery ST16 scanner; 2 iterations and 21 subsets for the Biograph mCT scanner, respectively). The values of axial spatial resolution at the center of the gantry were 4.80 (Discovery ST16 scanner) and 2.16 mm (Biograph mCT scanner), respectively.

### ^***18***^***F-FDG PET image analysis***

In accordance with previous studies in the field of head and neck malignancies [16, 20], a fixed SUVmax threshold of 40% (T40) was used for segmentation of mandibular lesions. A SUVmax threshold of 99.9% was applied to localize the SUVmax voxel; upon identification of this voxel of interest (VOI), we measured the mean Hounsfield Unit (HU) on the corresponding CT images. The SUVmax VOI was considered located with bone predominance when the mean CT HU was > 275 [21] in all other cases, a soft tissue predominance localization was assigned. Segmentation of mandibular lesions and SUVmax localization were accomplished by two experienced nuclear medicine physicians (N.-M.C. and T.-C.Y.) who were blinded to clinical and pathological data. All decisions were taken by consensus.

### Radiomics

PET radiomics features were extracted from VOI using the intensity histogram, gray-level co-occurrence matrix (GLCM), gray-level run-length matrix (GLRLM), and gray-level size zone matrix (GLSZM). A relative resampling method (64 bins) was applied to minimize noise that resulted from image processing [22]. PET radiomics parameters were calculated using the Chang-Gung Image Texture Analysis toolbox (CGITA) [23]. The terms and equations of PET texture parameters and the calculation procedures were consistent with the tenets set forth by the Imaging Biomarker Standardization Initiative (IBSI) [24].

### Statistical analysis

The associations between the study variables were determined by calculating Spearman’s correlation coefficients (*ρ*). Receiver operating characteristic (ROC) curve analysis was performed to select clinical variables and PET parameters associated with MRFS. All variables that produced an area under the ROC curve significantly different from 0.5 were included in subsequent analyses. The Youden’s statistic was used to determine the optimal cut-off points for variables associated with MRFS. Patients were dichotomized based on the identified cut-off values for subsequent survival analyses. MRFS curves were plotted using the Kaplan–Meier method and compared with log-rank tests. Independent predictors of MRFS were identified using univariate and multivariate Cox proportional hazards regression models. Schoenfeld residuals were applied to assess the proportional hazards assumption. To minimize overfitting during model construction, we relied on the general rule of thumb for multivariate analysis [25]. Results are expressed as hazard ratios (HRs) with 95% confidence intervals (CIs). On analyzing the predictive ability of different parameters, we compared the concordance index (*C*-index) of each variable using a nonparametric approach implemented in MedCalc, version 19.1 (Mariakerke, Belgium) [26]. All other data were analyzed using SPSS, version 16.0 (SPSS Inc., Chicago, IL, USA), with all tests two-sided at a 5% level of significance.

## Results

### General characteristics of the study patients

Table 1 shows the general characteristics of the 103 study participants. Most patients were men and had a positive history of risky oral habits—including tobacco smoking and alcohol drinking. The most common tumor site was buccal carcinoma followed by tongue carcinoma. Most patients presented with advanced T-stage disease, although nodal involvement was relatively limited. The majority of the study participants were treated with radical surgery followed by post-operative concurrent chemoradiotherapy (CCRT) for their primary cancer. The median radiation dose in the entire study cohort was 66 Gy (range 60–88 Gy)—with the radiation field including the jaw in all cases. Ninety-four cases had received postoperative RT or CCRT in this study. Among them, ninety cases (95.7%) had RT dose within the range of 60–66 Gy. Four patients (4.3%) who had margin positive or extranodular extension had received additional boost RT (total dose: 68–88 Gy). Nevertheless, the RT dose did not associate with mandible cancer recurrence (area under the ROC curve: 0.536, *P* = 0.620) in our study. At the time of PET imaging, 41 patients (39.80%) had evidence of cancer recurrence or a second primary head and neck malignancy.

**Table 1:** General characteristics of the study patients (*n* = 103) and univariate Cox regression analysis for mandibular relapse-free survival

Characteristic		*n* (%)	HR (95% CI)	*P*
Age at diagnosis	≤ 52 years	45 (43.69)	3.87 (1.59–9.42)	0.003
	> 52 years	58 (56.31)		
Sex	Female	5 (4.9)		
	Male	98 (95.1)	22.76 (0.02–34,393)	0.403
History of smoking	Yes	88 (85.44)	2.64 (0.61–11.37)	0.193
History of alcohol use	Yes	72 (69.90)	0.86 (0.38–1.97)	0.722
Diabetes	Yes	24 (23.30)	0.95 (0.35–2.55)	0.914
Cancer sites	Buccal	36 (34.95)	1.07 (0.46–2.46)	0.882
	Tongue	29 (28.16)		
	Gum	22 (21.36)		
	Mouth floor	8 (7.77)		
	Other sites*	8 (7.77)		
T stage	T1–T2	32 (31.07)		
	T3–T4	71 (68.93)	2.95 (0.87–9.98)	0.081
N stage	N-negative	51 (49.51)		
	N-positive	52 (50.49)	1.01 (0.45–2.27)	0.983
AJCC stage	I–II	16 (15.53)		
	III–IV	87 (84.47)	1.28 (0.38–4.35)	0.689
Primary treatment	Surgery plus CCRT	76 (73.79)	0.45 (0.19–1.06)	0.067
	Surgery plus RT	18 (17.48)		
	IC plus CCRT	7 (6.80)		
	CCRT	2 (1.94)		
SUV_max_ voxel site	Soft tissue predominance	60 (58.25)	5.08 (1.51–17.08)	0.009
	Bone predominance	43 (41.75)		
PET/CT parameters	SUVmax > 12.35	36 (34.95)	4.22 (1.74–10.23)	0.001
	SUVmax ≤ 12.35	67 (65.05)		
	TLG > 62.68	25 (24.27)	7.23 (3.08–16.98)	< 0.001
	TLG ≤ 62.68	78 (75.73)		

*HR* hazard ratio, *CI* confidence interval, *CCRT* concurrent chemoradiotherapy, *RT* radiotherapy, *IC* induction chemotherapy, *AJCC* American Joint Committee on Cancer, *SUVmax* maximum standardized uptake value, *TLG* total lesion glycolysis

*Other sites included hard palate (*n* = 2), lip (*n* = 2), soft palate (*n* = 2), and tonsils (*n* = 2)

### Mandibular cancer recurrence: associations with clinical and imaging parameters

Every patient underwent surgery for ORN or cancer recurrence in our study (12 patients underwent excisional biopsies; 65 cases underwent limited sequestrectomy and debridement; 10 patients received radical sequestrectomy and flap reconstruction; 16 ones underwent cancer-wide excision). The results of histopathology revealed that mandibular cancer recurrence occurred in 24 (23.30%) study participants (eight cases were proved by excisional biopsies; two patients by limited sequestrectomy and debridement; 14 ones by wide excision). Two patients underwent surgery of wide excision, but the pathological reports revealed only ORN.

The median follow-up time was 48.0 months (interquartile range: 31.5–67.4 months) in the entire cohort. For patients with ORN and mandibular recurrences, the follow-up time was 18.4 months and 26.5 months with corresponding interquartile ranges of 9.6–42.6 and 13.1–73.4 months, respectively. Recurrent subgroup tended to have longer follow-up time than ORN one with marginal significance (*P* = 0.109). However, the follow-up time could not differentiate ORN from recurrence (area under the ROC curve: 0.616, *P* = 0.087). On univariate Cox regression analysis (Table 1), an age at onset ≤ 52 years was associated with an increased risk of mandibular cancer recurrence (*P* = 0.003). Associations of borderline statistical significance were observed for T3–T4 disease and treatment with radical surgery followed by post-operative CCRT. Mandibular cancer recurrence did not show significant associations with other clinical variables.

On analyzing PET parameters, a voxel site of SUVmax with soft tissue predominance, increased total lesion glycolysis (TLG), and elevated SUVmax values were all significantly associated with an increased likelihood of mandibular cancer recurrence (Table 1). ROC curve analyses of TLG, SUVmax, and other radiomics parameters are presented in Additional file 1: Table S1. Patients with mandibular cancer recurrence had higher TLG (104.34 ± 111.42 g vs. 38.86 ± 28.87 g, respectively, *P* < 0.001) and SUVmax values (15.45 ± 7.62 vs. 9.80 ± 3.67, respectively, *P* < 0.001) compared with those without. Upon calculation of the maximum Youden’s indices, TLG and SUVmax values were dichotomized according to their optimal cut-off values (TLG > 62.68 vs. ≤ 62.68; SUVmax > 12.35 vs. ≤ 12.35).

A TLG > 62.68 g was not significantly correlated with either age ≤ 52 years (*ρ* = 0.05, *P* = 0.622) or a voxel site of SUVmax (*ρ* = 0.07, *P* = 0.508). However, significant correlations were observed for a SUVmax > 12.35 (age ≤ 52 years: *ρ* = 0.216, *P* = 0.028; voxel site of SUVmax with soft tissue predominance, *ρ* = 0.249, *P* = 0.011). A significant correlation was also noted between a TLG > 62.68 g and a SUVmax > 12.35 (*ρ* = 0.297, *P* = 0.002).

### Predictors of mandibular recurrence-free survival

**Fig. 1:**
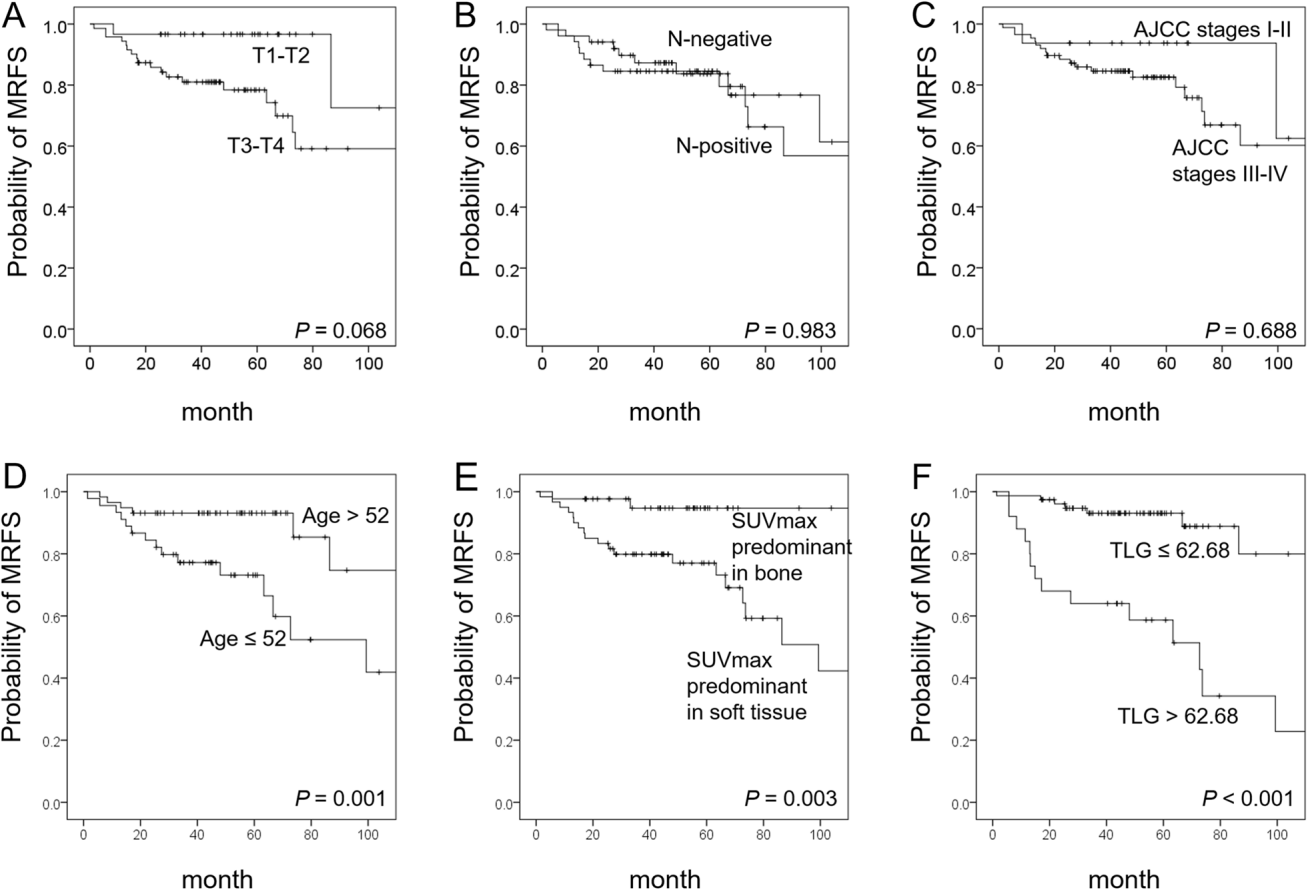
Kaplan–Meier plots of mandibular relapse-free survival (MRFS) rates in patients with OSCC stratified according to T stage (**A**), N stage (**B**), AJCC stage (**C**), age at cancer diagnosis (**D**), SUVmax site (**E**), and TLG (**F**). The Youden’s statistic was used to determine the optimal cut-off point for each variable. *P* values according to log-rank tests are presented in the insets

Kaplan–Meier plots of MRFS according to different clinical and PET parameters are reported in Fig. 1. Less favorable MRFS was observed for patients with an age at diagnosis ≤ 52 years, a voxel site of SUVmax with soft tissue predominance, and a TLG > 62.68 g. Because of the collinearity between SUVmax and TLG, these parameters were entered separately into multivariate analysis (Model 1 and Model 2, respectively; Table 2). The results revealed that a TLG > 62.68 g, an age at diagnosis ≤ 52 years, and a voxel site of SUVmax with soft tissue predominance were independent adverse prognostic factors for MRFS.

**Table 2:** Multivariable Cox regression analysis of mandibular relapse-free survival

Variable	HR (95% CI)	*P*
*Model 1*		
Age at diagnosis ≤ 52 years	3.36 (1.31–8.64)	0.012
Voxel site of SUVmax with soft tissue predominance	5.02 (1.45–17.31)	0.011
SUVmax > 12.35	2.38 (0.94–6.06)	0.068
*Model 2*		
Age at diagnosis ≤ 52 years	3.36 (1.30–8.73)	0.013
Voxel site of SUVmax with soft tissue predominance	4.35 (1.27–14.90)	0.019
TLG > 62.68 g	5.38 (2.24–12.91)	< 0.001

*HR* hazard ratio, *CI* confidence interval, *SUVmax* maximum standardized uptake value, *TLG* total lesion glycolysis

### Prognostic scoring system for the prediction of mandibular recurrence-free survival

Finally, we devised a prognostic scoring system for the prediction of MRFS based on the three independent adverse prognostic factors identified from multivariate analysis (0 for the absence and 1 for the presence). The following distribution of risk scores was observed in the study cohort: score 0, *n* = 18; score 1, *n* = 50; score 2; *n* = 25; and score 3, *n* = 10. Mandibular cancer recurrence was observed in 0 (0%), 3 (6%), 12 (48%), and 9 (90%) patients with a score of 0, 1, 2, and 3, respectively. Patients with score of 2–3 were considered at high risk, whereas those with a score of 0 or 1 were a low-risk group. High-risk patients had a significantly higher likelihood of mandibular cancer recurrence (HR 32.50, 95% CI 8.51–124.18, *P* < 0.001).

**Fig. 2:**
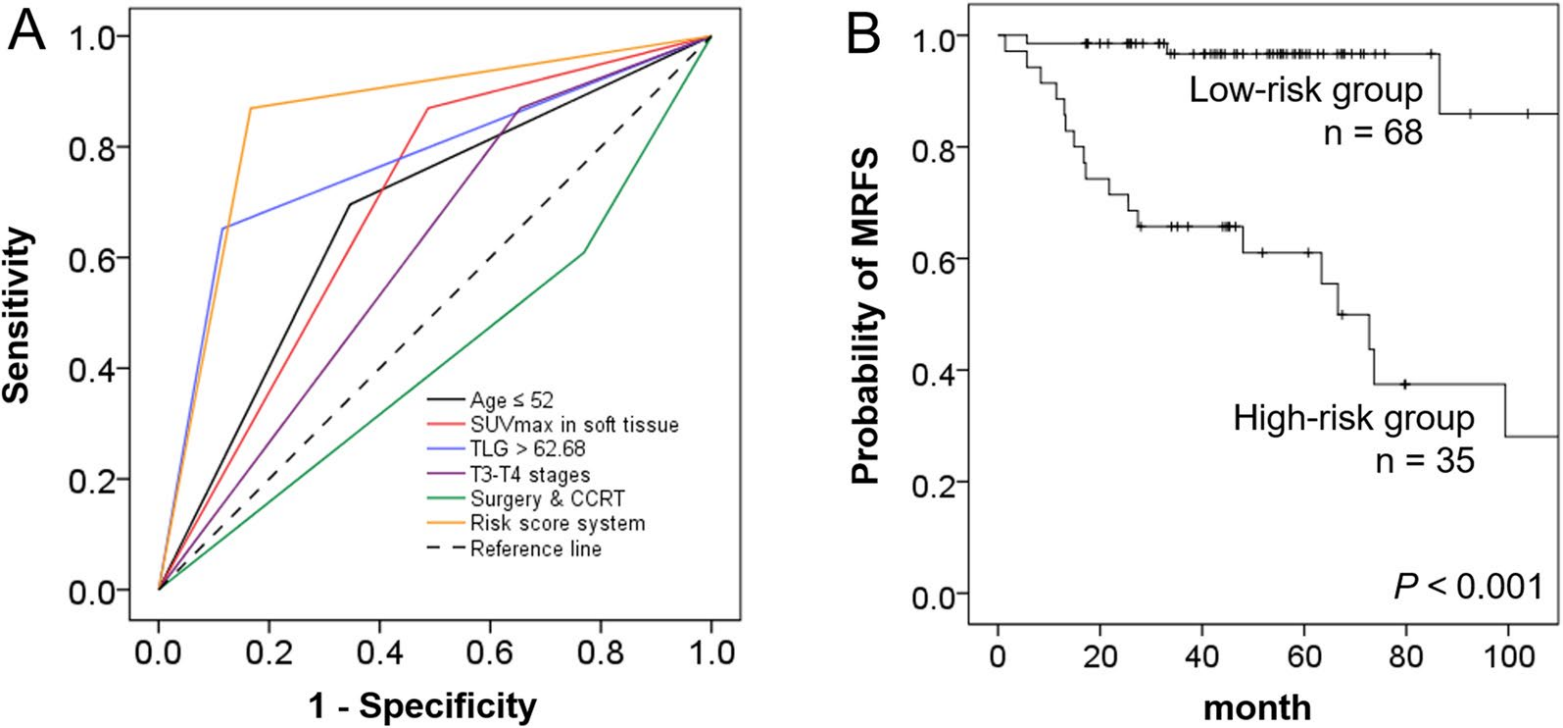
Receiver operating characteristic curve analyses and *C-*indices for the prediction of MRFS (**A**). The *C*-index of the simple scoring system was higher than those calculated for other parameters. Kaplan–Meier plots of MRFS in patients with OSCC classified as being at low- versus high-risk according to the simple scoring system (**B**). *P* values according to log-rank tests are presented in the insets

**Fig. 3:**
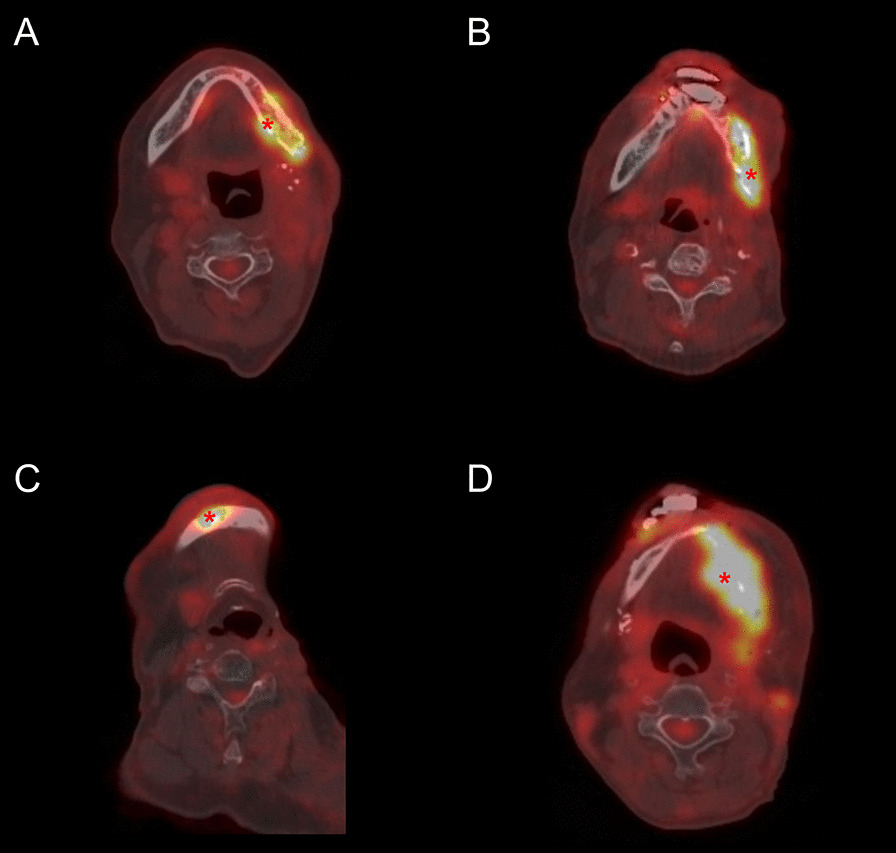
PET/CT image **A** obtained from a patient with left buccal cancer (T3N2bM0; AJCC stage IVA) diagnosed at 59 years of age. The patient had an elevated mandibular TLG (149.28 g, see main text), but the voxel site of SUVmax (asterisk) was located with bone predominance (CT HU: 340.0). A score of 1 was assigned. The results of histopathology revealed the presence of mandibular osteoradionecrosis (ORN). PET/CT image **B** obtained from a patient with left buccal cancer (T3N1M0; AJCC stage III) diagnosed at 56 years of age. The patient had an elevated mandibular TLG (73.66 g) and showed a voxel site of SUVmax located with soft tissue predominance (asterisk) (CT HU: 186.0). A score of 2 was assigned. The results of histopathology revealed the presence of mandibular cancer recurrence. PET/CT image **C** obtained from a patient with left buccal cancer (T3N2bM0; AJCC stage IVA) diagnosed at 67 years of age. The patient had a low mandibular TLG (51.27 g) and showed a voxel site of SUVmax located with soft tissue predominance (asterisk) (CT HU: 97.2). A score of 1 was assigned. The results of histopathology revealed the presence of mandibular ORN. PET/CT image **D** obtained from a patient with a score of 3. He was diagnosed with left lower gum cancer (T2N0M0, stage II) at 45 years of age. The patient had an elevated mandibular TLG (175.47 g) and showed a voxel site of SUVmax located with soft tissue predominance (asterisk) (CT HU: 18.0). The results of histopathology revealed the presence of mandibular cancer recurrence

The scoring system had a sensitivity of 87.50%, a specificity of 82.28%, and an overall accuracy of 83.50% for identifying mandibular cancer recurrence. As Fig. 2 shows, the scoring system (*C*-index = 0.85) outperformed several parameters—including T3–T4 disease (*C*-index = 0.59, *P* < 0.001), treatment with surgery and post-operative CCRT (*C-*index = 0.57, *P* < 0.001), age at diagnosis ≤ 52 years (*C*-index = 0.68, *P* = 0.002), a voxel site of SUVmax within soft tissue (*C*-index = 0.69, *P* < 0.001), and high TLG values (*C*-index = 0.73, *P* = 0.052)—for the prediction of MRFS. Figure 3 shows illustrative PET images obtained in high- versus low-risk patients.

## Discussion

The clinical outcomes of patients with OSCC who had undergone primary multidisciplinary treatment remain heterogeneous [27]. Although recent years have witnessed significant technical advances in the field of RT techniques, mandibular ORN remains a significant clinical concern. Here, we demonstrate that a simple scoring system—based on the presence of a mandibular TLG > 62.68 g, an age at diagnosis ≤ 52 years, and a voxel site of SUVmax located with soft tissue predominance—was clinically useful for identifying patients at high risk of mandibular cancer recurrence. In addition, the scoring system provided a reliable stratification of MRFS in patients with OSCC.

This is, to our knowledge, the first study to provide a comprehensive analysis of clinical variables and PET imaging parameters in relation to the risk of mandibular cancer recurrence in patients with suspected ORN of the jaw. As far as PET variables are concerned, we found that TLG—a parameter which provides information regarding both lesion volume and metabolic activity—outperformed the predictive value of SUVmax for mandibular cancer recurrence. It is possible that the high collinearity of mandible SUVmax with age at diagnosis and the SUVmax voxel site could have attenuated its clinical significance in the prediction of mandibular cancer recurrence. Another interesting finding from our study is the predictive value of a voxel site of SUVmax located with soft tissue predominance, which is in accordance with the study by Meerwein et al. [11]. It is well-known that cancer recurrences tend to occur in soft tissues characterized by abundant vascular networks rather than in poorly vascularized bone structures. However, malignant cells located in close proximity to osseous tissue may activate osteoclastogenesis and ultimately promote both bone resorption and cancer dissemination [28, 29]. Although this may offer an explanation for the observed association between a voxel site of SUVmax located within soft tissue and mandibular cancer recurrence, further mechanistic studies are warranted.

Differently from TLG and SUVmax, PET radiomics parameters did not show significant associations with MRFS in our study. While the exact underlying reasons remain unclear, it is possible that the use of two different CT attenuation maps (i.e., bone and soft tissue) during PET image reconstruction of mandibular lesions might have played a role. Accordingly, there is evidence that an elevated noise and image misregistration may lead to inaccurate estimation of radiomics parameters [24, 30, 31].

The impact of patient age on the clinical outcomes of OSCC is a matter of ongoing debate [32]. In our study, an age at diagnosis ≤ 52 years was identified as an adverse prognostic factor for MRFS. It has been previously reported that young patients with OSCC tend to show a higher immunohistochemical expression of p53 in malignant tissues [33]—which in turn represents an unfavorable prognostic biomarker [34–37]. Our findings may prompt additional investigations on the association between p53 expression and the occurrence of mandibular cancer recurrence in OSCC.

The scoring system devised in our study may have major clinical implications for the differential diagnosis of mandibular lesions in patients with OSCC who had undergone primary RT. In general, the following strategy can be applied for patients with suspected ORN of the jaw following RT. Since mandibular cancer recurrence occurred very rarely in low-risk patients (4.41%), biopsy can be avoided and traditional surgical management for ORN of the jaw should be sufficient. However, high-risk patients require second-level diagnostic procedures—including histopathological analysis of biopsy samples—for ruling out the presence of mandibular cancer recurrence. Further research is necessary to examine the clinical appropriateness of the proposed scoring system.

There are limitations to this study. First, this is a single-center investigation and the results clearly require replication. The retrospective nature of this study is prone to unavoidable confounding and residual confounding and a selection bias cannot be excluded. Second, our research was specifically focused on PET parameters and the potential diagnostic value of CT imaging patterns was not taken into account. However, the significance of an infiltrative growth pattern on CT images is not univocal and may reflect either the presence ORN [12] or tumor recurrence [11]. Finally, we acknowledge that lesion segmentation and the localization of SUVmax site were based partly on visual interpretation—which is prone to inter-observer variability.

## Conclusion

The scoring system described in our study may be clinically useful for identifying mandibular cancer recurrence in patients with OSCC and suspected ORN of the jaw. High-risk patients may benefit from a diagnostic biopsy for ruling out the presence of tumor recurrence, whereas the use of histopathology can be avoided in those with a score of 0 or 1. Our findings should be considered as hypothesis-generating and require validation in independent clinical cohorts.

## Supplementary Information

**Additional file 1.** Supplementary Table 1.

## Data Availability

The datasets used and/or analyzed during the current study are available from the corresponding author on reasonable request.
