# Supplementary material for: The added values of 18F-FDG PET/CT in differentiating cancer recurrence and osteoradionecrosis of mandible in patients with treated oral squamous cell carcinoma
Source: EJNMMI Res. 2023 Apr 3;13:25. doi: 10.1186/s13550-023-00965-8 (PMC10070584; doi:10.1186/s13550-023-00965-8)
Supplement: Supplementary file 1 — Additional file 1. Supplementary Table 1. [file 13550_2023_965_MOESM1_ESM.doc]

**SUPPLEMENTARY MATERIALS**

**The Added Values of ^18^F-FDG PET/CT in Differentiating Cancer Recurrence and Osteoradionecrosis of Mandible in Patients with**

**Treated Oral Squamous Cell Carcinoma**

| **Supplementary Table 1** PET radiomics parameters for the prediction of mandibular relapse-free survival. | | | |
| --- | --- | --- | --- |
| Matrix | Parameter | ROC curve analysis | |
|  |  | AUC (range) | *P* |
| Intensity histogram | SUVmax | 0.747 (0.636−0.858) | < .001 |
| Intensity histogram | SUV Skewness | 0.371 (0.217−0.525) | .056 |
| Intensity histogram | SUV Kurtosis | 0.573 (0.447−0.700) | .278 |
| Intensity histogram | TLG | 0.697 (0.546−0.848) | .004 |
| Intensity histogram | Metabolically active tumor volume | 0.589 (0.441−0.737) | .187 |
| Intensity histogram | Entropy | 0.468 (0.321−0.614) | .634 |
| Intensity histogram | Surface area | 0.613 (0.464−0.762) | .095 |
| Intensity histogram | Asphericity | 0.624 (0.494−0.754) | .067 |
| GLCM | Second angular moment | 0.513 (0.363−0.664) | .845 |
| GLCM | Contrast | 0.458 (0.320−0.597) | .538 |
| GLCM | Entropy | 0.502 (0.351−0.654) | .975 |
| GLCM | Homogeneity | 0.599 (0.473−0.725) | .142 |
| GLCM | Dissimilarity | 0.449 (0.31−0.589) | .454 |
| GLCM | Inverse difference moment | 0.611 (0.486−0.737) | .100 |
| GLCM | Correlation | 0.506 (0.375−0.637) | .932 |
| GLRLM | Short run emphasis | 0.460 (0.329−0.592) | .558 |
| GLRLM | Long run emphasis | 0.541 (0.410−0.672) | .543 |
| GLRLM | Intensity variability | 0.573 (0.438−0.707) | .282 |
| GLRLM | Run-length variability | 0.578 (0.432−0.724) | .248 |
| GLRLM | Run percentage | 0.418 (0.284−0.553) | .227 |
| GLRLM | Low-intensity run emphasis | 0.487 (0.356−0.619) | .851 |
| GLRLM | High-intensity run emphasis | 0.521 (0.378−0.663) | .761 |
| GLRLM | Low-intensity short-run emphasis | 0.448 (0.314−0.582) | .440 |
| GLRLM | High-intensity short-run emphasis | 0.521 (0.385−0.657) | .755 |
| GLRLM | Low-intensity long-run emphasis | 0.531 (0.399−0.663) | .645 |
| GLRLM | High-intensity long-run emphasis | 0.501 (0.356−0.645) | .994 |
| GLSZM | Short-zone emphasis | 0.466 (0.332−0.600) | .612 |
| GLSZM | Large-zone emphasis | 0.563 (0.428−0.698) | .351 |
| GLSZM | Intensity variability | 0.617 (0.477−0.757) | .084 |
| GLSZM | Size-zone variability | 0.528 (0.385−0.672) | .674 |
| GLSZM | Zone percentage | 0.372 (0.251−0.493) | .058 |
| GLSZM | Low-intensity zone emphasis | 0.488 (0.340−0.637) | .864 |
| GLSZM | High-intensity zone emphasis | 0.495 (0.349−0.641) | .938 |
| GLSZM | Low-intensity short-zone emphasis | 0.479 (0.338- 0.620) | .755 |
| GLSZM | High-intensity short-zone emphasis | 0.487 (0.345−0.630) | .851 |
| GLSZM | Low-intensity large-zone emphasis | 0.504 (0.362−0.645) | .956 |
| GLSZM | High-intensity large-zone emphasis | 0.557 (0.408−0.706) | .399 |
| Abbreviations: ROC, receiver operating characteristic; AUC, area under curve; GLCM, gray level co-occurrence matrix; GLRLM, gray-level run-length matrix; GLSZM: gray-level size zone matrix. | | | |
